# Metabolic Effects of Breaking Prolonged Sitting With Standing or Light Walking in Older South Asians and White Europeans: A Randomized Acute Study

**DOI:** 10.1093/gerona/gly252

**Published:** 2018-11-07

**Authors:** Thomas Yates, Charlotte L Edwardson, Carlos Celis-Morales, Stuart J H Biddle, Danielle Bodicoat, Melanie J Davies, Dale Esliger, Joe Henson, Aadil Kazi, Kamesh Khunti, Naveed Sattar, Alan J Sinclair, Alex Rowlands, Latha Velayudhan, Francesco Zaccardi, Jason M R Gill

**Affiliations:** 1 Diabetes Research Centre, College of Life Sciences, University of Leicester, Australia; 2 NIHR Leicester Biomedical Research Centre, Leicester General Hospital, University Hospitals of Leicester NHS Trust, Australia; 3 Institute of Cardiovascular and Medical Sciences, University of Glasgow, Australia; 4 Institute for Resilient Regions, University of Southern Queensland, Springfield Central, Australia; 5 School of Sport, Exercise, and Health Sciences, Loughborough University, Birmingham; 6 National Centre for Sport and Exercise Medicine, University of Loughborough, Diabetes Frail Ltd and University of Aston, Birmingham; 7 Leicester Diabetes Centre, University Hospitals of Leicester NHS Trust, Diabetes Frail Ltd and University of Aston, Birmingham; 8 NIHR Collaborations for Leadership in Applied Health Research and Care (CLAHRC) East Midlands, Diabetes Frail Ltd and University of Aston, Birmingham; 9 Foundation for Diabetes Research in Older People, Diabetes Frail Ltd and University of Aston, Birmingham; 10 Institute of Psychiatry, Psychology and Neurosciences, King’s College London; 11 Department of Health Sciences, University of Leicester

**Keywords:** Ethnicity, Physical activity, Sedentary behavior, Walking

## Abstract

**Background:**

Prolonged sitting is common in older adults and is associated with insulin resistance and poor cardiometabolic health. We investigate whether breaking prolonged sitting with regular short bouts of standing or light walking improves postprandial metabolism in older white European and South Asian adults and whether effects are modified by ethnic group.

**Methods:**

Thirty South Asian (15 women) and 30 white European (14 women) older adults (aged 65–79 years) undertook three experimental conditions in random order. (a) *Prolonged sitting*: continuous sitting during an observation period if 7.5 hours consuming two standardized mixed meals. (b) *Standing breaks*: sitting interrupted with 5 minutes of standing every 30 minutes (accumulating 60 minutes of standing over the observation period). (c) *Walking breaks*: sitting interrupted with 5 minutes of self-paced light walking every 30 minutes (accumulating 60 minutes of walking). Blood samples (glucose, insulin, triglycerides) and blood pressure were sampled regularly throughout each condition.

**Results:**

Compared with prolonged sitting, walking breaks lowered postprandial insulin by 16.3 mU/L, (95% CI: 19.7, 22.0) with greater reductions (*p* = .029) seen in South Asians (22.4 mU/L; 12.4, 32.4) than white Europeans (10.3 mU/L; 5.9, 14.7). Glucose (0.3 mmol/L; 0.1, 0.5) and blood pressure (4 mm Hg; 2, 6), but not triglycerides, were lower with walking breaks, with no ethnic differences. Standing breaks did not improve any outcome.

**Conclusions:**

Breaking prolonged sitting with short bouts of light walking, but not standing, resulted in clinically meaningful improvements in markers of metabolic health in older adults, with South Asians gaining a greater reduction in postprandial insulin.

**Trial Registration:**

NCT02453204

## Introduction

Over half of older adults have type 2 diabetes or nondiabetic hyperglycemia (1, 2), with up to 90% having hypertension (3). The increased prevalence of dysglycemia in older age results from declining insulin sensitivity, which is a hallmark of ageing and a risk factor for cognitive impairment and frailty (4, 5). Within industrialized Western countries, ageing-related declines in insulin sensitivity are further accelerated within some minority ethnic groups, particularly South Asians where the risk of type 2 diabetes is over twice that seen in white European populations (6, 7). Pragmatic interventions that improve insulin sensitivity in older adults across different ethnicities are therefore needed.

In modern society, time spent engaged in sedentary behaviors, defined as sitting or reclining with low energy expenditure, accounts for over half the waking day (8). Observational studies have shown that low levels of sedentary time are associated with a lower risk of mortality and morbidity within the general population along with healthy ageing, better physical function, and greater health-related quality of life in older age (9–14). Interventions focusing on reducing sedentary time have therefore been highlighted as a priority research area for the promotion of healthy ageing (15). Experimental studies have shown that breaking prolonged sitting with regular bouts of light walking improves postprandial insulin sensitivity (16), with some studies suggesting that breaking prolonged sitting simply by standing may also be beneficial (17). However, the generalizability of these findings to older adults or nonwhite ethnic groups has not been investigated.

The aim of this study was to investigate the acute (short-term) effect on postprandial metabolic responses of regularly breaking prolonged sitting with short bouts of standing or self-paced light walking in older adults and to investigate whether these effects differ between white European and South Asian adults.

## Methods

### Design

This study was a multisite (Leicester and Glasgow, United Kingdom) randomized three condition crossover trial (design displayed in Supplementary Figure 1). The three conditions were the following: (a) continuous prolonged sitting, (b) sitting with standing breaks of 5 minutes every half hour (60 minutes of standing in total) and (c) sitting with light self-paced walking breaks of 5 minutes every half hour (60 minutes of light walking in total; Supplementary Figure 1). Each condition was carried out over a period of 7.5 hours within laboratories at the Universities of Leicester and Glasgow. Two standardized mixed meals were provided during each condition. A minimum washout of 7 days between conditions was used. Postprandial insulin was defined a priori as the primary outcome.

Informed consent was obtained from all eligible participants, and ethics approval was provided from an NHS Research Ethics Committee (Derby, UK).

### Participants

This trial aimed to recruit an equal number of white Europeans and South Asians and an equal number of men and women within each ethnicity. South Asian ethnicity was defined as anyone identifying themselves as Asian or Asian British (Indian, Pakistani, Bangladeshi, or other). Participants were recruited from May 2015 to November 2016 across two sites (Leicester and Glasgow) through invitation from existing data sets, attending community events for older people and minority ethnic groups, and strategic placement and distribution of promotional materials. The study was completed in December 2016. Inclusion criteria were adults aged between 65 and 79 years inclusive, able to walk, able to communicate in and understand English, and able to provide informed consent. Exclusion criteria were undertaking regular purposeful exercise (≥75 minutes of self-reported vigorous exercise per week), having a psychological or neurological condition that limits participation in the study (eg, dementia), steroid use, and use of glucose-lowering medication.

Supplementary Figure 2 shows the flow of study recruitment. In total, 76 were consented, 70 individuals were randomized of which 60 were included in the study (15 South Asian women, 15 South Asian Men, 14 white European women, and 16 white European men).

### Familiarization and Baseline Visit

All participants visited their study center for a familiarization and screening visit. Participants were checked for eligibility, consented and shown the location and facilities where the intervention would take place. Body mass, waist circumference (midpoint between the lower costal margin and iliac crest), and height were measured to the nearest 0.1 kg, 0.5 cm, and 0.5 cm, respectively. Smoking status and medical history were collected by interview.

Participants were asked to complete handgrip and sit-to-stand tests to assess physical function. Handgrip strength was measured by hydraulic dynamometer (Jamar, Pennsylvania) with participants seated with their elbow by their side and arm at 90° with a neutral forearm and wrist position. Three measurements were taken, with the second and third measurement used to calculate an average for each hand. The sit-to-stand test was administered using a standard height chair without arm rests. Participants were instructed to rise to a full standing position and return to a seated position with their arms crossed as many times as possible within 60 seconds. The 1 minute sit-to-stand test is an established measure of physical function that correlates with other measures of physical fitness, such the 6 minutes walking test (18).

A blood test was taken for the measurement of total and high-density lipoprotein cholesterol.

In the 7 days following familiarization, participants were asked to wear a thigh mounted triaxial accelerometer (activPAL, PAL Technologies, Glasgow, Scotland) to record habitual sitting, standing, and stepping time, respectively. ActivPAL accelerometers have been shown to have good validity for measuring time spent in sitting, standing, and walking activity (19, 20). Data were downloaded using the manufacturer’s software (activPAL Professional Research Edition, PAL Technologies, Glasgow, UK) and processed using a validated automated algorithm in STATA (StataCorp LP), which defines valid and invalid wear days based on achieving certain criteria, such as having at least 10 hours of wear time and achieving at least 500 steps (21). For the purposes of this study, at least one valid day was required.

### Randomization

After familiarization and baseline data were collected, allocation to one of six possible intervention sequences was generated using a randomization service (Sealed Envelope https://www.sealedenvelope.com/) by the Leicester Clinical Trials Unit, stratified by center, ethnicity, and sex. Biomedical data were analyzed blinded to treatment allocation.

### Experimental Procedures

The experimental regimens are highlighted in Supplementary Figure 1.

#### Measurement structure across each experimental regimen

Participants were asked to arrive at the laboratory following an overnight fast of 12 hours. Participants were also instructed to avoid any alcohol for 2 days and any vigorous exercise for 3 days prior to each experimental condition. In order to measure compliance with the physical activity restriction, participants were requested to wear a wrist-mounted accelerometer (Original GENEActiv monitors, Activinsights, Cambridgeshire) in the period between each condition. GENEActiv accelerometers can be used to measure overall physical activity volume as well as time spent in moderate-to-vigorous physical activity (22, 23); analysis methods and data are presented in Supplementary Table 1).

Participants were also asked to record all food and drink consumed in the 48 hours before the first experimental condition in a food diary and to replicate this diet before the remaining two experimental conditions.

Participants arrived at the laboratory by motorized transport, had a cannula inserted into an arm, and sat quietly for 60 minutes. A fasting blood sample was then taken, which was followed by a standardized mixed-meal breakfast consisting of 8 kcal/kg of body weight, with a macronutrient composition reflective of a typical Western diet (13% protein, 52% carbohydrate, and 35% fat). Participants were allowed 15 minutes to complete the meal. Blood was sampled at 30, 60, 120, and 180 minutes postprandially. A standardized lunch identical to breakfast was then consumed. Further blood samples were taken at 30, 60, 120, and 180 minutes after lunch. Prior to each blood sample, blood pressure (two measurements a minute apart), affect (Feeling Scale, ranging from very good [+5], to neutral [0], to very bad [−5]), and daytime sleepiness (Karolinska Sleepiness Scale, ranging from extremely alert [+1] to neither alert or sleepy (+5) to extremely sleepy, fighting sleep [+9]) were also assessed (24, 25). The research staff supervised participants throughout each study condition to ensure full compliance with the trial protocols. Participants consumed water ad libitum during the first of the experimental conditions and were then asked to replicate the volume ingested in subsequent conditions.

#### Sitting condition

Participants remained seated throughout the test period of 7.5 hours while undertaking typical sedentary behaviors such as watching TV, using a computer, reading, and writing. Walking and standing were restricted.

#### Standing condition

Participants were instructed to break their sitting time by standing close to their chair for 5 minutes, 15 minutes after breakfast and every 30 minutes thereafter until lunch (6 standing bouts). The standing protocol then resumed 15 minutes after lunch and every 30 minutes thereafter (6 bouts). Participants were asked to stand in the same position with no further instructions provided. In total, 12 bouts (60 minutes) of standing were undertaken throughout the test period of 7.5 hours.

#### Walking condition

Participants were instructed to break their sitting time with 5 minutes of self-paced light walking, 15 minutes after breakfast and every 30 minutes thereafter until lunch (6 bouts). The walking protocol then resumed 15 minutes after lunch and every 30 minutes thereafter (6 bouts). In total, 12 bouts (60 minutes) of walking were undertaken throughout the test period. Participants were instructed to walk up and down a marked track in the laboratory at a pace they felt was comfortable and of light intensity. Walking speeds ranged from 2.4 to 4.4 km/h.

### Biochemical Analysis

All fasted and postprandial venous blood samples were collected into K2 ethylenediaminetetraacetic acid (EDTA) tubes, placed immediately on ice, and centrifuged to separate plasma within 15 minutes. Plasma was stored at −80°C for later analysis. All samples were analyzed within the same location (University of Glasgow) using the same procedure. Glucose and triglycerides across each experimental condition, along with lipid profile at familiarization, were analyzed using clinically validated automated biochemistry platform (c311, Roche Diagnostics, Burgess Hill, UK). Insulin was measured with an equivalent immunoassay platform (e411, Roche Diagnostics, Burgess Hill, UK). The analyzers were calibrated and quality controlled using the manufacturer’s materials. Coefficient of variation over two levels of controls was less than 3% for biochemistry assays and less than 6% for insulin.

The homeostasis model of insulin resistance (HOMA-IR) was calculated as fasting insulin level (mU/L) × fasting glucose (mmol/L)/22.5. HOMA-IR has been shown to correlate well with the euglycemic clamp in nondiabetic populations (*r* > .7) and has been reported to have reasonable reproducibility (26).

### Sample Size

Allowing for a 20% change in insulin area under the curve (AUC) for walking or standing compared with sitting (27), a standardized difference of 1, an intraindividual correlation of 0.3, and an alpha level of 0.05, we required at least 56 participants (28 South Asian, 28 white Europeans) to complete the trial. This sample size achieved more than 90% power for a main effect of treatment within each ethnicity and 80% power for an overall treatment × ethnicity interaction assuming a change in postprandial insulin concentrations that are twice as great in one ethnic group compared to the other.

### Data Inclusion

Participants with a minimum of 50% of scheduled blood samples across each condition were included; 6 individuals (8.6% of those randomized) were removed with missing data due to an inability to cannulate (Supplementary Figure 2). In the included sample, there were a total of 25 (1.7%) missing data points for the primary outcome due to incomplete blood sampling out of a possible 1,440 postprandial blood samples collected. Missing data were imputed using a regression method reported previously for acute experimental studies (17, 28); this approach uses key predictors (body mass index, ethnicity, age, fasting values, and treatment condition) to derive a regression equation for the insulin, glucose, and triglyceride values at each time point.

### Data Analysis

Time-averaged AUC (calculated using the trapezium rule) was used as a summary measure for postprandial insulin, glucose, triglyceride, and blood pressure responses with values representing the average level over the measurement period. AUC_glucose_ × AUC_insulin_ was used as an insulin resistance index as described previously (29); for ease of interpretation, values were normalized to the mean level during the sitting condition for white Europeans. Generalized estimating equations with an exchangeable correlation matrix were used to analyze the data, taking into account repeated measures across treatments. Due to right skewed distributions of positive values, insulin and triglycerides data were analyzed using a gamma distribution with an identity link. Interaction terms were fitted to test whether the effect of treatment was modified by ethnicity or sex. All models were adjusted for age and fasting value, the latter to take account of any differences between conditions and ethnicities. Treatment effects were investigated by comparing the standing and walking conditions to prolonged sitting.

For the primary outcome, significant treatment × ethnicity or treatment × sex interactions were followed by sensitivity analyses to (a) adjust the model for HOMA-IR rather than fasting insulin to further explore whether interactions were independent of differences in insulin resistance, (b) further adjust for the number of 60 seconds sit-to-stand repetitions to investigate whether interactions were independent of differences in physical function.

Results are reported as mean (95% CI) unless stated otherwise; *p* less than .05 was considered significant. Data were analyzed in SPSS version 24.

## Results

Sixty individuals were included for analysis (South Asian = 30, white European = 30) (see Supplementary Figure 2); participant characteristics are shown in Table 1. South Asian participants had higher levels of insulin resistance (HOMA-IR = 2.25 [interquartile range (IQR) 1.51, 3.46]) than white Europeans (HOMA-IR = 1.47 [IQR 0.97, 2.54]). Participants from both ethnic groups also had elevated systolic blood pressure (142 mm Hg [IQR 132, 155]) with a minority taking blood pressure (25%) or lipid-lowering (12%) medication. On the basis of a median of 7 days of free living data collection with activPAL accelerometers, participants spent the majority of their waking day sitting (60%), with only 11% spent in any form of stepping activity.

**Table 1.** Participant Characteristics

Characteristics	All (*n* = 60)	South Asian (*n* = 30)	White European (*n* = 30)
Age (years)	70.0 (67, 75)	69 (66, 75)	71 (67, 76)
BMI (kg/m^2^)	26.6 (24.9, 28.3)	26.7 (23.7, 29.5)	26.5 (25.0, 28.3)
Waist circumference (cm)	94.0 (86.0, 100.0)	95.5 (84.5, 98.0)	92.0 (87.0, 98.0)
Sex (female)	29 (48)	15 (50)	14 (47)
Handgrip strength (kg)	24.4 (19.1, 32.4)	25.5 (19.6, 31.6)	22.1 (18.4, 32.6)
Sit-to-stand (repetitions during 60 s)	23 (19, 27)	21 (19, 26)	24 (21, 27)
Smoke			
Never	44 (73)	28 (93)	16 (53)
Past	14 (23)	1 (3)	13 (43)
Current	2 (3)	1 (3)	1 (2)
Blood pressure medication	15 (25)	9 (30)	6 (20)
Lipid-lowering medication	7 (12)	6 (20)	1 (3)
Systolic blood pressure (mm Hg)	142 (132, 155)	142 (136, 158)	142 (121, 156)
Diastolic blood pressure (mm Hg)	75 (70, 83)	74 (67, 84)	76 (73, 83)
Total cholesterol (mmol/L)	4.6 (3.8, 5.5)	4.4 (3.8, 5.1)	4.7 (3.8, 5.6)
HDL cholesterol (mmol/L)	1.4 (1.1, 1.7)	1.3 (1.1, 1.5)	1.6 (1.2, 1.9)
Fasting insulin (mU/L)^†^	8.6 (5.0, 12.6)	11.0 (7.6, 13.4)	6.6 (4.6, 11.8)
Fasting glucose (mmol/L)^†^	5.1 (4.6, 5.5)	5.1 (4.7, 5.6)	5.0 (4.4, 5.5)
HOMA-IR	1.81 (1.11, 2.86)	1.48 (0.97, 2.54)	2.25 (1.51, 3.46)
Triglycerides (mmol/L)^†^	0.9 (0.6, 1.1)	1.1 (0.8, 1.4)	0.7 (0.5, 0.9)
Time spent sedentary^‡^ (h/d)	9.0 (7.6, 9.8)	9.3 (8.2, 10.0)	8.8 (6.9, 9.7)
Time Spent standing^‡^ (h/d)	4.4 (4.0, 5.4)	4.6 (4.1, 5.4)	4.4 (3.9, 5.4)
Time spent stepping^‡^ (h/d)	1.7 (1.4, 2.2)	1.6 (1.3, 1.9)	2.0 (1.5, 2.4)

Note: Continuous data shown as median (interquartile range) and categorical data as number (%). BMI = body mass index; HDL = high-density lipoprotein; HOMA-IR = homeostasis model of insulin resistance.

^†^Average fasting value across the three experimental conditions.

^‡^Data missing for five South Asians and one white European. South Asians reported a median of 15.6 hours per day of waking wear time over a median of 7 days, with white Europeans reporting 15.4 hours per day of waking wear over 7 days.

### 

#### Primary outcome

**Figure 1.**
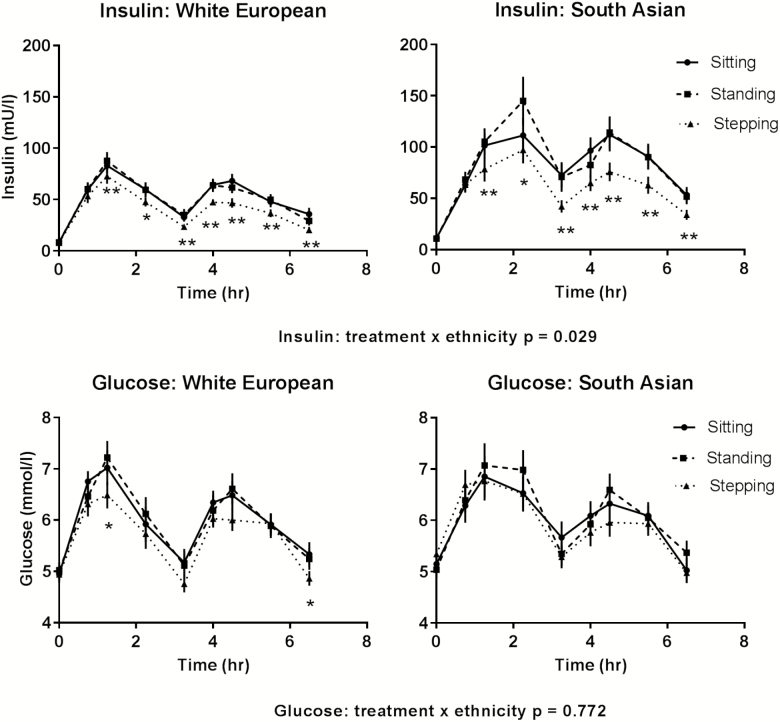
Postprandial insulin and glucose responses across each condition for South Asians and white Europeans. Error bars show the standard error within each condition at each time point. *p < .05, **p < .01 for the walking breaks compared to prolonged sitting.

Insulin responses were 16.3 (19.7, 22.0) mU/L lower during walking breaks compared to prolonged sitting with responses modified by ethnicity (*p* = .029; see Table 2), but not sex (*p* = .516). For South Asians, the insulin response was reduced by 22.4 (12.4, 32.4) mU/L (27%) during walking breaks compared to prolonged sitting, whereas for white Europeans there was a 10.3 (5.9, 14.7) mU/L (19%) reduction. There was no intervention effect for standing breaks compared with prolonged sitting (Table 2). Postprandial insulin responses for treatment varied across time (*p* < .001) with differences seen for walking, but not standing, breaks compared with prolonged sitting at most time points for both ethnicities, with the greatest reduction occurring at 60 minutes after lunch (Figure 1).

**Table 2.** Postprandial Responses for Insulin, Glucose, and Triglycerides During Each Treatment Condition

	White European			South Asian					
Variable	Prolonged sitting	Standing Breaks	Walking Breaks	Prolonged sitting	Standing Breaks	Walking Breaks	*p* for treatment	*p* for ethnicity	*p* for ethnicity × treatment
Insulin (mU/L)	54.6 (46.1, 63.1)	55.6 (46.6, 64.5)	44.3 (38.1, 50.6)**	83.6 (65.9, 101.3)	85.6 (68.8, 102.4)	61.2 (50.5, 71.9)**	<.001	.003	.029
Glucose (mmol/L)	6.1 (5.7, 6.5)	6.1 (5.7, 6.4)	5.8 (5.5, 6.0)*	6.1 (5.7, 6.5)	6.2 (5.9, 6.6)	5.9 (5.5, 6.2)	<.001	.625	.772
Triglycerides (mmol/L)	1.1 (1.1, 1.2)	1.2 (1.1, 1.3)	1.2 (1.1, 1.3)	1.3 (1.3, 1.4)	1.4 (1.3, 1.5)*	1.3 (1.3, 1.4)	.002	.006	.110
Systolic blood pressure (mm Hg)	128 (125, 131)	128 (125, 130)	123 (120, 127)**	128 (125, 131)	127 (124, 131)	125 (122, 128)*	.003	.925	.808

Note: Data adjusted for age, fasting value, and sex and displayed as time-averaged response (95% CI).

*p < .05 compared to sitting control within each ethnicity.

**p < .01 compared to sitting control within each ethnicity.

Results were not altered if the model was adjusted for HOMA-IR rather than fasting insulin or after further adjustment for physical function (Supplementary Table 2).

#### Secondary outcomes

Glucose responses were 0.3 (0.1, 0.5) mmol/L lower during walking breaks compared to prolonged sitting; responses across treatment conditions were not modified by ethnicity (*p* = .772, Table 2) or sex (*p* = .204). There was no effect for standing breaks (Table 2). Postprandial glucose responses for treatment varied across time (*p* < .001; (Figure 1)

**Figure 2.**
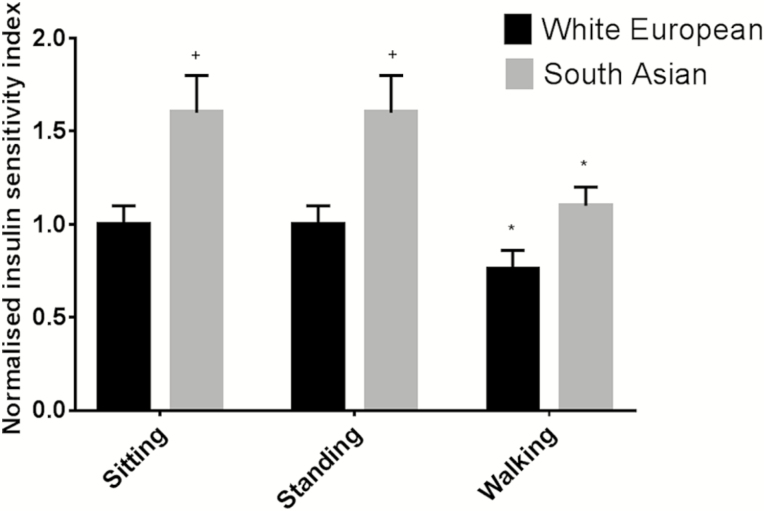
Insulin resistance index within each condition for South Asians and white Europeans. Data displayed as normalized scores (*SE*), representing the fold difference compared to white Europeans during prolonged sitting. *p < .05 compared to prolonged sitting within each ethnic group. +*p* < .05 compared to white Europeans during prolonged sitting.

For the insulin resistance index, there was an effect for treatment (*p* < .001) with values during walking breaks reduced by 27% (16%, 39%); responses across treatment conditions were not modified by sex (*p* = .901) or ethnicity (*p* = .104). Figure 2 displays normalized values for each condition stratified by ethnicity.

Triglyceride responses were 0.1 (0.0, 0.2) mmol/L higher during the standing breaks condition compared to the sitting, with no effect for walking breaks; responses across treatment conditions were not modified by ethnicity (*p* = .110, Table 2) or sex (*p* = .303). Supplementary Figure 3 shows the response over time for each ethnic group.

Systolic blood pressure was 4 mm Hg (2, 6) lower during walking breaks compared to prolonged sitting; responses across treatment conditions were not modified by ethnicity (*p* = .808, Table 2) or sex (*p* = .420). There was no effect for standing breaks (Table 2). Supplementary Figure 4 shows the response over time for ethnic group. There was no effect for diastolic blood pressure.

For daytime sleepiness, there was a significant effect for treatment (*p* = .025) with sleepiness lowest during walking breaks (Supplementary Figure 5); responses across treatment conditions were not modified by ethnicity (*p* = .217) or sex (*p* = .067). Participants reported a positive affective state throughout each condition, with no difference between conditions (Supplementary Figure 6).

Accelerometer analyses revealed there was no difference in overall physical activity volume or time spent in purposeful moderate–to-vigorous physical activity over a median to 6 days leading up to each condition (Supplementary Table 1).

## Discussion

This study found that breaking prolonged sitting with regular 5 minute bouts of self-paced light walking reduced postprandial insulin, glucose, and blood pressure in a cohort of white Europeans and South Asian older adults. The reduction in the primary outcome of postprandial plasma insulin was greater for South Asians, where responses were reduced by 22.4 mU/L compared to 10.3 mU/L in white Europeans after adjusting for differences in fasting insulin or insulin resistance. By contrast, breaking prolonged sitting with regular bouts of static standing did not improve any outcome in either South Asians or white Europeans.

As populations continue to live longer, healthy ageing is a public health priority. Insulin resistance is a key marker of ageing and a risk factor for cardiometabolic disease, cognitive impairment, and frailty (4, 30–32). A previous study has shown that a 20% difference in insulin AUC following an oral glucose tolerance test is associated with a 10% difference in coronary mortality risk (33), suggesting the effect of walking breaks on postprandial insulin observed in this study for South Asians (27% reduction) and white Europeans (19% reduction) was clinically meaningful. Systolic blood pressure also increases with age and is associated with an elevated risk of cardiovascular disease (34), with an intensive intervention to lower systolic blood pressure in older adults shown to reduce the risk of a cardiovascular event by 25% (35). The difference in daytime blood pressure between the prolonged sitting and walking breaks conditions seen in this study (4 mm Hg) has been associated with a 6% difference in the risk of cardiovascular mortality (36). This study therefore suggests breaking prolonged sitting time with regular short bouts of light movement may be an effective strategy for inducing clinically meaningful reductions in insulin resistance and blood pressure in older adults. Most public health guidelines for older adults have predominantly focused on the promotion of moderate-intensity physical activity in bouts of at least 10 minutes (37). However, the majority of older adults fail to engage in regular purposeful physical activity (38, 39). Focusing on breaking prolonged sitting time with short periods of light-intensity physical activity could therefore represent an alternative or complementary strategy for health promotion.

As far as we are aware, this is the first study to investigate ethnic differences in the response to breaking prolonged sitting. South Asians have a higher risk of cardiometabolic disease than white Europeans and are therefore a priority group for disease prevention (6). The increased risk of cardiometabolic disease in South Asians has been linked to several mechanisms, including those related to insulin sensitivity (6). This was emphasized in the current study where measures of postprandial insulin and insulin resistance were higher in South Asians than white Europeans. Our findings are consistent with previous observational and experimental research, which has suggested the association between moderate-to-vigorous intensity physical activity and metabolic health may be greater in South Asians or those with a higher risk of type 2 diabetes (40–42). Our experimental study extends these observations by confirming that although the reduction in postprandial insulin was greater in South Asians compared with white Europeans, light walking was insufficient to fully attenuate the difference between ethnicities.

The self-paced walking breaks employed in this study ranged from 2.4 to 4.4 km/h, indicating an intensity of movement within the capability of the vast majority of older adults. The cardiometabolic benefit seen with employing light walking breaks in white Europeans and South Asians extends previous findings in the general and high-risk populations (16,17, 43). However, our study did not find a benefit of reducing prolonged sitting with regular standing breaks, suggesting at least some movement is required to gain metabolic benefit in older adults. This finding is consistent with other studies in younger healthy populations but in contrast to a study in women with dysglycemia (16, 17). The discrepancy between these studies could be explained by the level of insulin resistance within the populations tested. We also cannot exclude the potential that longer periods of standing may be effective but these are likely to be less pragmatically applied in real-life settings.

A key strength is that this is the first experiential study to the best of our knowledge designed to test the acute effect of breaking prolonged sitting in older adults and whether effects are different in South Asians compared with white Europeans or men compared to women. However, there are also several limitations. This study tested the acute effect over a single day. Further research is needed to explore the chronic effect of reducing and breaking prolonged bouts of sitting over the longer term in free-living rather than laboratory-based conditions. We acknowledge that although testing the effect of breaking sitting every 30 minutes throughout the day may be a suitable approach for some, more ecologically valid approaches taking into account typical daily activity patterns also need to be tested to inform later phase behavior change interventions. Finally, although we tested postprandial responses following a meal that was typical of Western diets, the magnitude of the glucose, insulin, and lipid responses to walking breaks are not necessarily generalizable to different dietary patterns.

In conclusion, breaking prolonged sitting with regular bouts of light walking, but not standing, reduced postprandial plasma insulin, glucose, and systolic blood pressure levels in a bi-ethnic population of older adults, with beneficial effects on postprandial insulin largest in South Asians who had higher levels of insulin resistance compared with white Europeans. Further research is needed to investigate the efficacy and effectiveness of interventions to reduce prolonged sitting in community and lifestyle interventions aimed at promoting healthy ageing in older adults from multiethnic communities.

## Funding

The research was supported by the UK Research Councils’ Lifelong Health and Wellbeing Initiative in partnership with the Department of Health (grant number MR/K025090/1); the National Institute for Health Research (NIHR) Leicester Biomedical Research Centre; the National Institute for Health Research Collaboration for Leadership in Applied Health Research and Care–East Midlands (NIHR CLAHRC–EM); and the Leicester Clinical Trials Unit.

## Conflict of Interest

None declared.

## Supplementary Material

gly252_suppl_Supplementary_Material
