# Supplementary material for: Metabolic Effects of Breaking Prolonged Sitting With Standing or Light Walking in Older South Asians and White Europeans: A Randomized Acute Study
Source: J Gerontol A Biol Sci Med Sci. 2018 Nov 7;75(1):139–46. doi: 10.1093/gerona/gly252 (PMC6909896; doi:10.1093/gerona/gly252)
Supplement: gly252_suppl_Supplementary_Material [file gly252_suppl_supplementary_material.docx]

**Metabolic effect of breaking prolonged sitting with standing or light walking in older South Asians and White Europeans: a randomized acute study**

Supplementary material

Contents

[Supplementary Table 1: Physical activity level prior to each condition 2](#_Toc516738055)

[Supplementary Table 2: Condition effects for insulin area under the curve further adjusted for physical function 3](#_Toc516738056)

[Supplementary Figure 1: Trial design 4](#_Toc516738057)

[Supplementary Figure 2: Participant flow 5](#_Toc516738058)

[Supplementary Figure 3: Postprandial triglyceride responses across each condition 6](#_Toc516738059)

[Supplementary Figure 4: Systolic blood pressure responses across each condition 7](#_Toc516738060)

[Supplementary Figure 5: Daytime sleepiness across each condition 8](#_Toc516738061)

[Supplementary Figure 6: Feeling scale responses across each condition 9](#_Toc516738062)

## Supplementary Table 1: Physical activity level prior to each condition

|  | White European | | | South Asian | | |
| --- | --- | --- | --- | --- | --- | --- |
|  | Sitting (n = 24) | Standing (n = 21) | Walking (n = 29) | Sitting (n = 26) | Standing (n = 26) | Walking (n = 27) |
| Wear days | 6 (5, 6) | 6 (5,6) | 6 (6, 7) | 6 (5,6) | 6 (5,6) | 6 (5,6) |
| ENMO (mg) | 24.4 (22.1, 26.7) | 23.6 (21.7, 25.5) | 24.6 (22.7, 26.5) | 25.0 (22.3, 27.7) | 25.3 (22.7, 28.0) | 25.0 (22.1, 28.0) |
| Purpose moderate or vigorous physical activity (mins/day) | 16.5 (10.2, 22.8) | 20.4 (11.0, 29.4) | 20.7 (13.4, 28.1) | 8.9 (4.3, 13.6) | 7.7 (3.8, 11.7) | 7.2 (3.8, 10.7) |

Data as mean (95% CI). p > 0.05 for all pairwise comparisons between conditions within each ethnicity

Individuals with at least one valid day (16 hours of wear) were included. GENEActiv .bin files were analysed with R-package GGIR version 1.2-0 (http://cran.r-project.org). Total physical activity volume was measured using the Euclidean norm minus 1g (ENMO) metric (measured in milligravity [mg] units) and purposeful physical activity was defined as 10 min of consecutive 5-s epochs where 80% of epochs were equal to, or higher than, 100 mg

## Supplementary Table 2: Condition effects for insulin area under the curve further adjusted for physical function

| Variables |  | White European | | | South Asian | | |  |  |  |
| --- | --- | --- | --- | --- | --- | --- | --- | --- | --- | --- |
| Model | Variable | Sitting | Standing Breaks | Walking Breaks | Sitting | Standing Breaks | Walking Breaks | P for treatment | P for ethnicity | P for ethnicity x treatment |
| Sensitivity analysis 1 | Insulin AUC (mU/l∙hr) | 55.0 (46.4, 63.5) | 55.6 (46.7, 64.5) | 43.9 (37.2, 50.6)** | 83.3 (65.7, 100.9) | 85.6 (69.1, 102.0) | 61.6 (50.4, 72.8)** | <0.001 | 0.004 | 0.023 |
| Sensitivity analysis 2 | Insulin AUC (mU/l∙hr) | 56.1 (47.7, 64.6) | 56.7 (48.1, 65.3) | 46.2 (39.7, 52.8)** | 82.1 (65.2, 99.2) | 84.3 (68.1, 100.6) | 59.7 (40.1, 70.3)** | <0.001 | 0.006 | 0.013 |

Sensitivity analysis 1: Adjusted for age, HOMA-IR and sex.

Sensitivity analysis 2: Adjusted for age, fasting level, sit to stand repetitions and sex.

## Supplementary Figure 1: Trial design


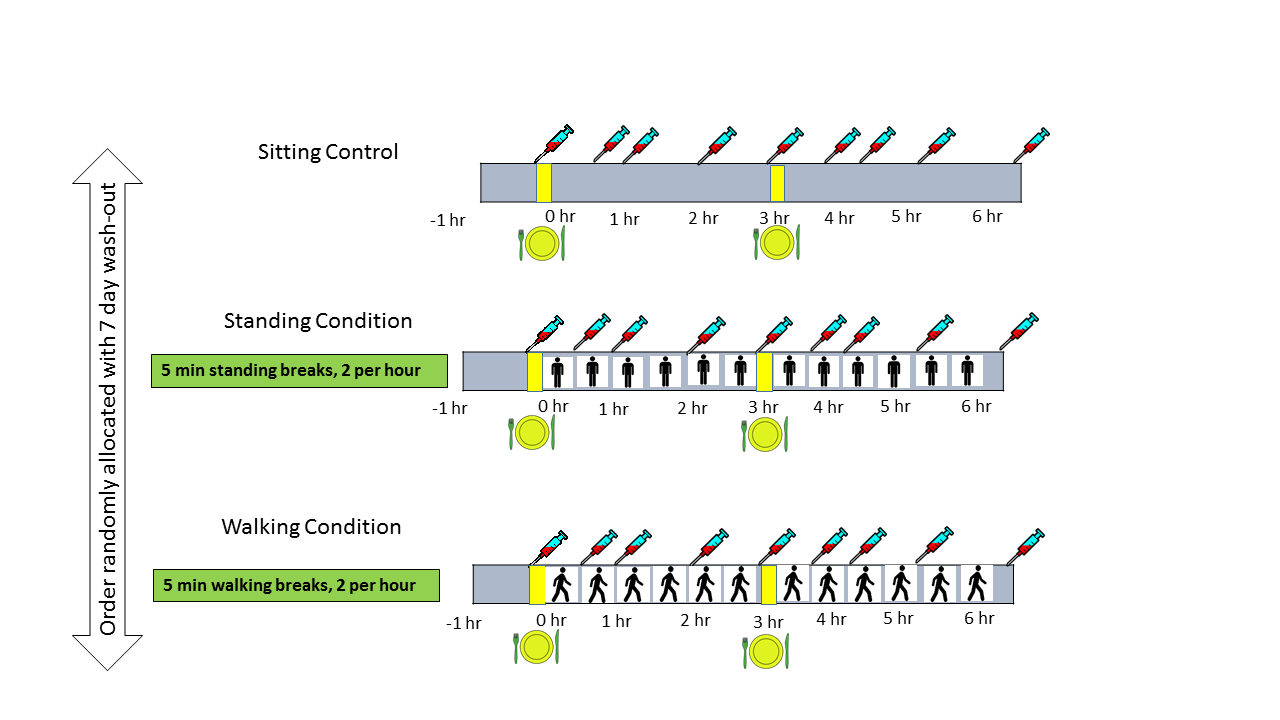


## Supplementary Figure 2: Participant flow

Number of community engagement activities undertaken = 21

Number of invitations sent = 331

Number of replies received = 122

- Willing to take part (n = 101)
- Not willing to take part (n = 21)

Number contacted = 101

Not Not meeting inclusion criteria (n = 16)

Unable to take part for other reasons (n = 11)

Number consented = 76

Ineligible (n = 6)

Number randomised = 70

Could not cannulate (n = 6)

Did not tolerate diet (n = 2)

Unable to commit time (n = 2)

Number included for data analysis = 60

## Supplementary Figure 3: Postprandial triglyceride responses across each condition


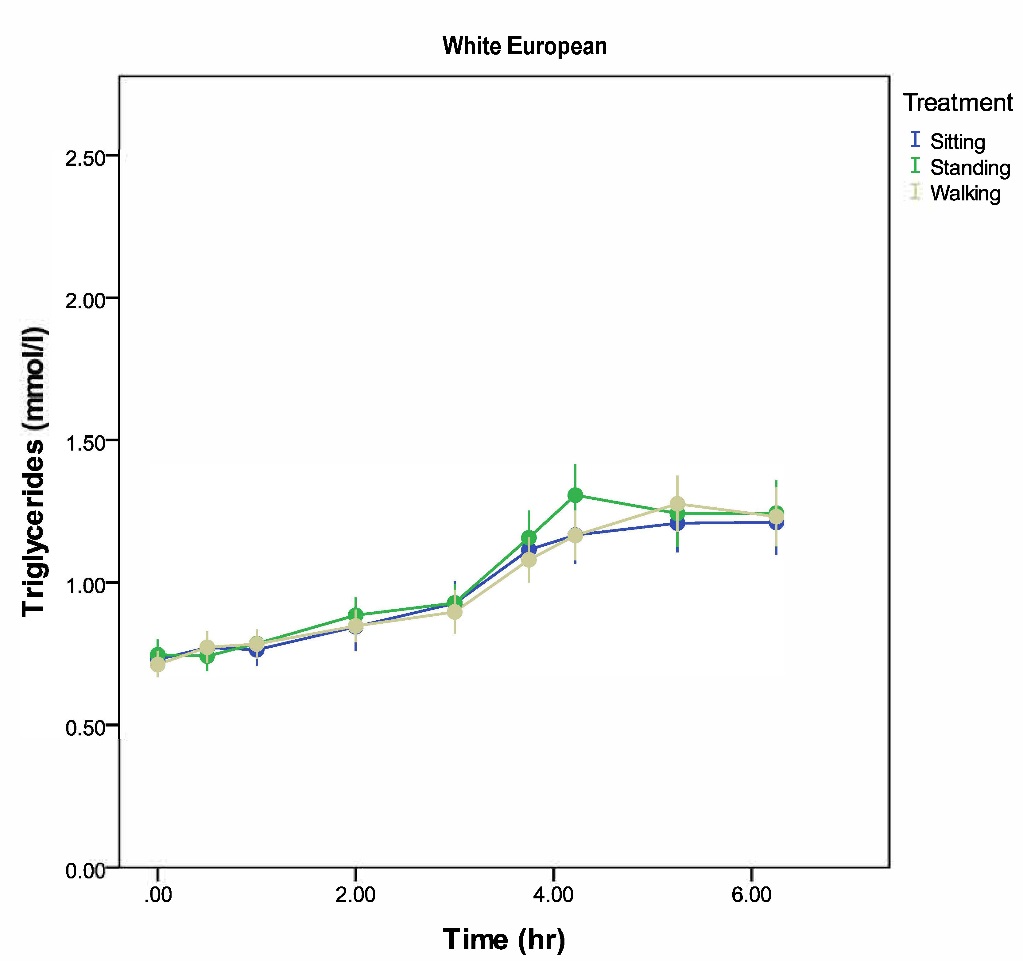

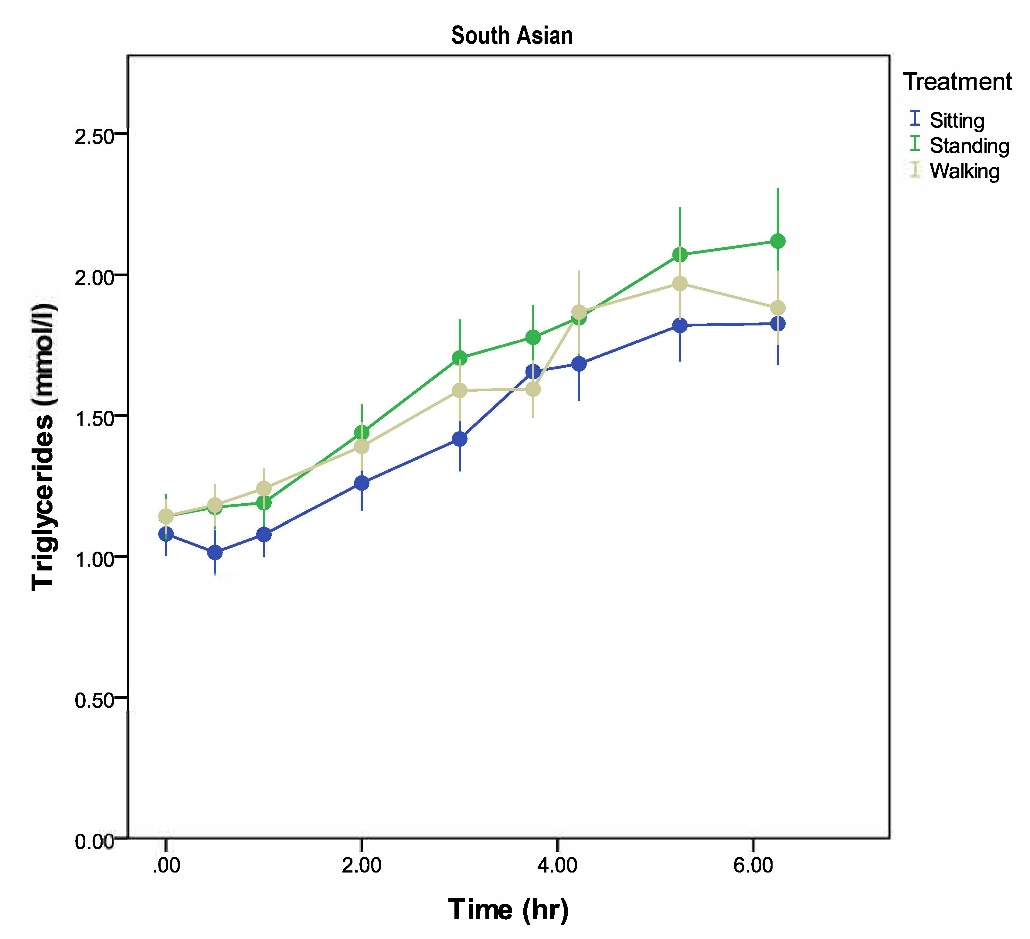


Error bars show the standard error within each condition at each time point

## Supplementary Figure 4: Systolic blood pressure responses across each condition


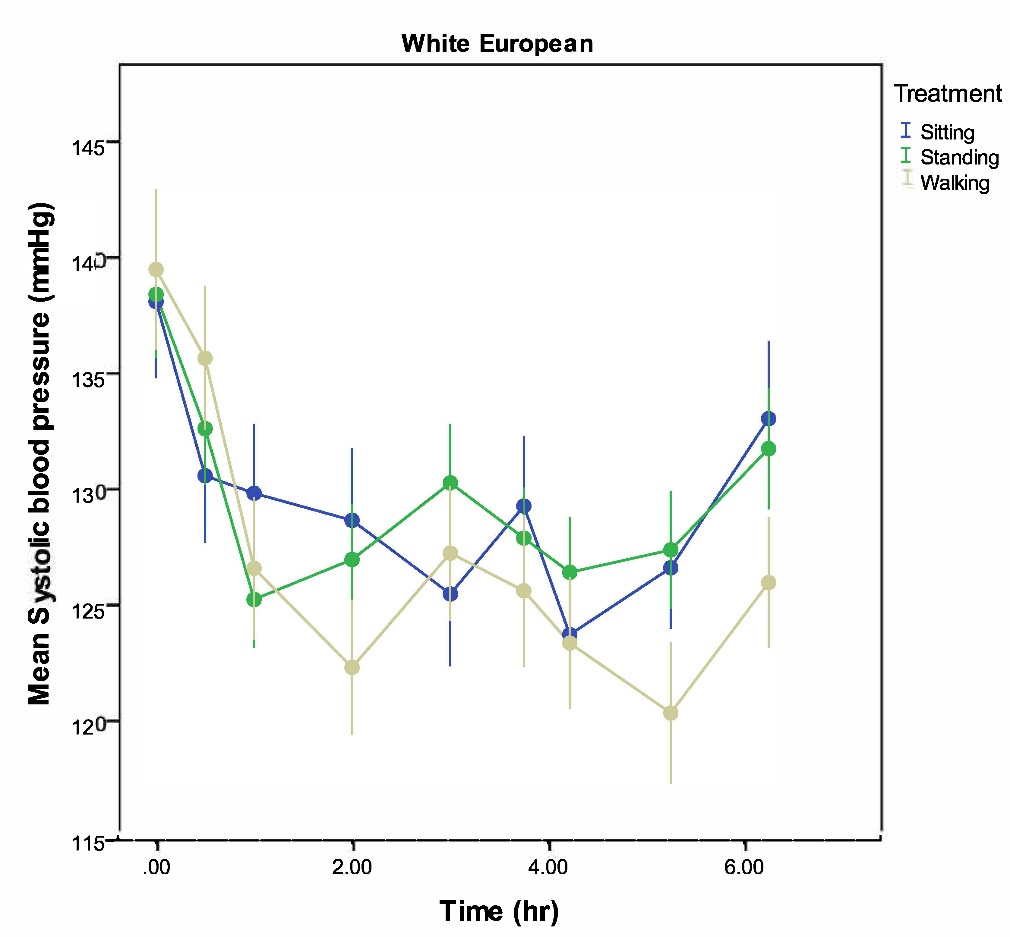

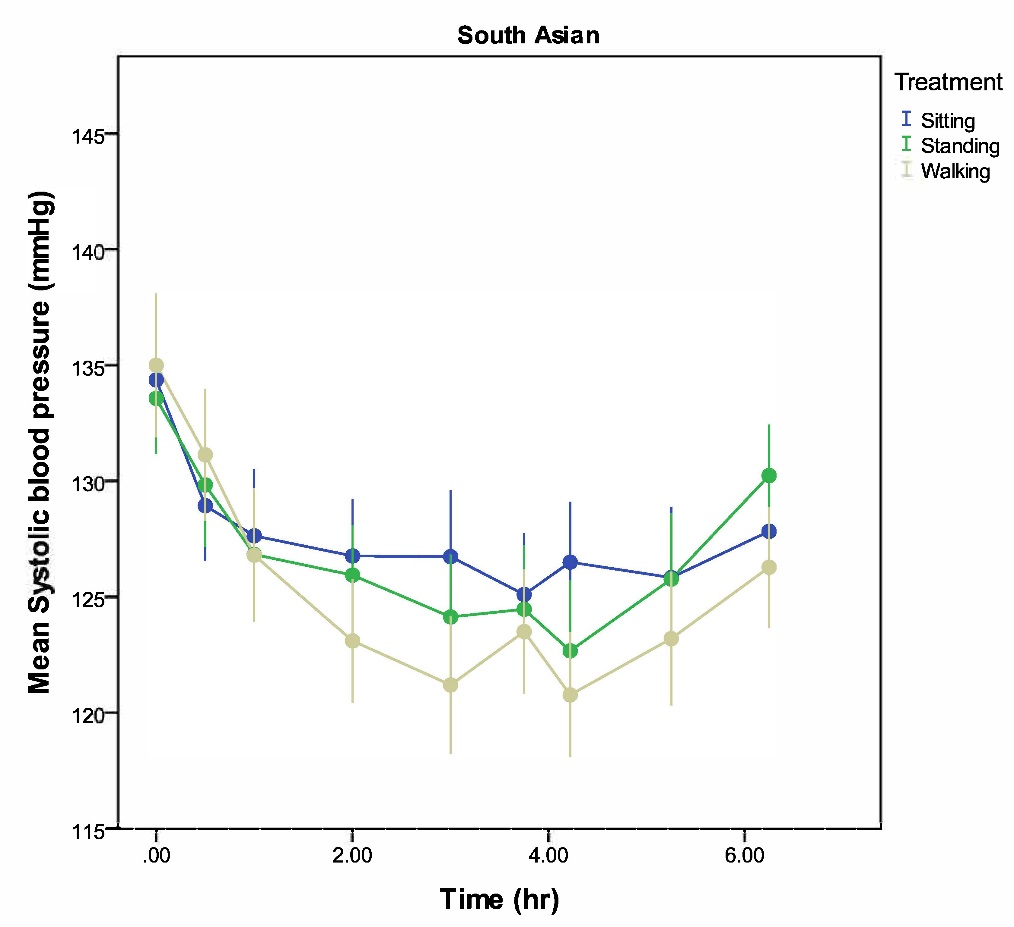


Error bars show the standard error within each condition at each time point

## Supplementary Figure 5: Daytime sleepiness across each condition


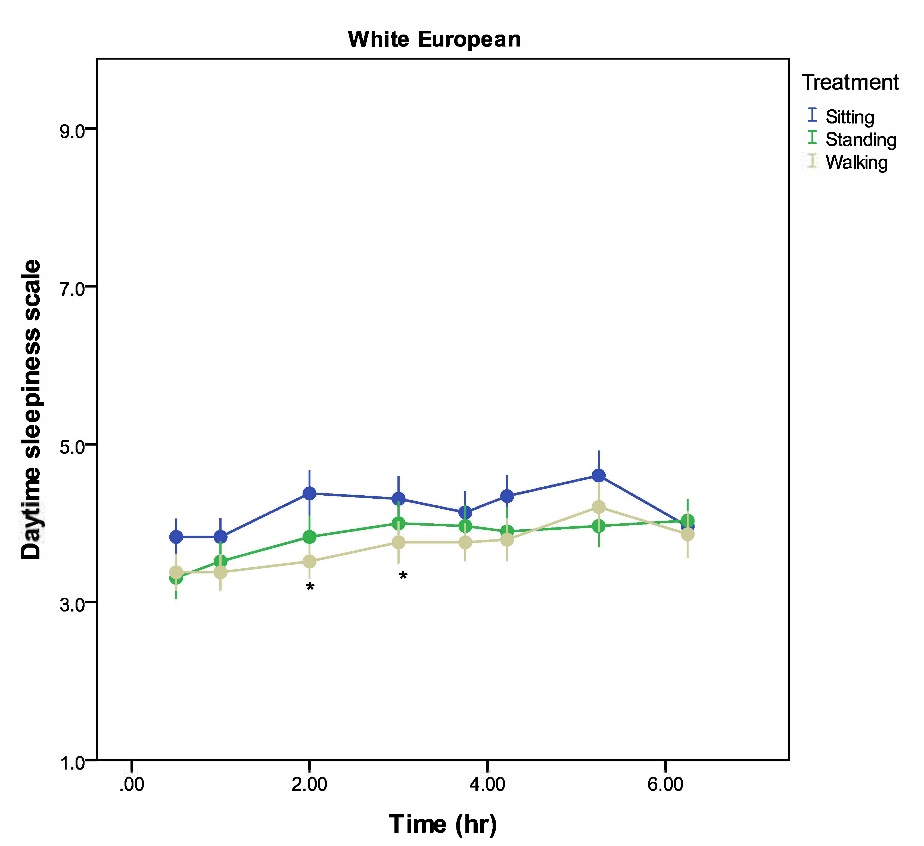

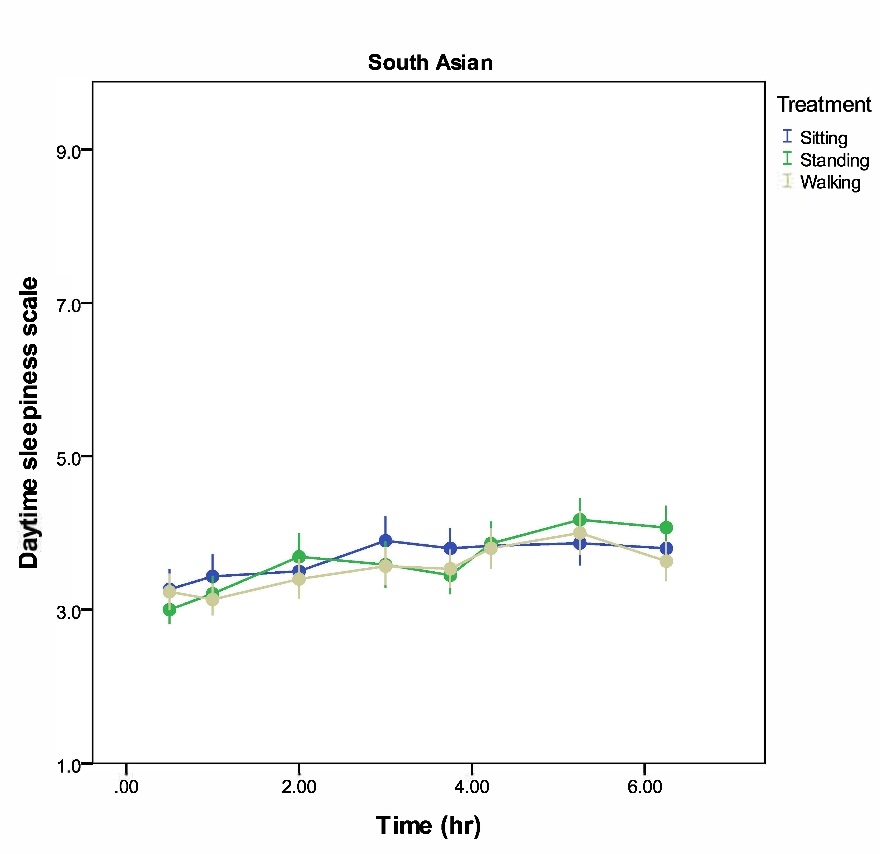


* = P < 0.05 for walking breaks compared to prolonged sitting

Error bars show the standard error within each condition at each time point

## Supplementary Figure 6: Feeling scale responses across each condition


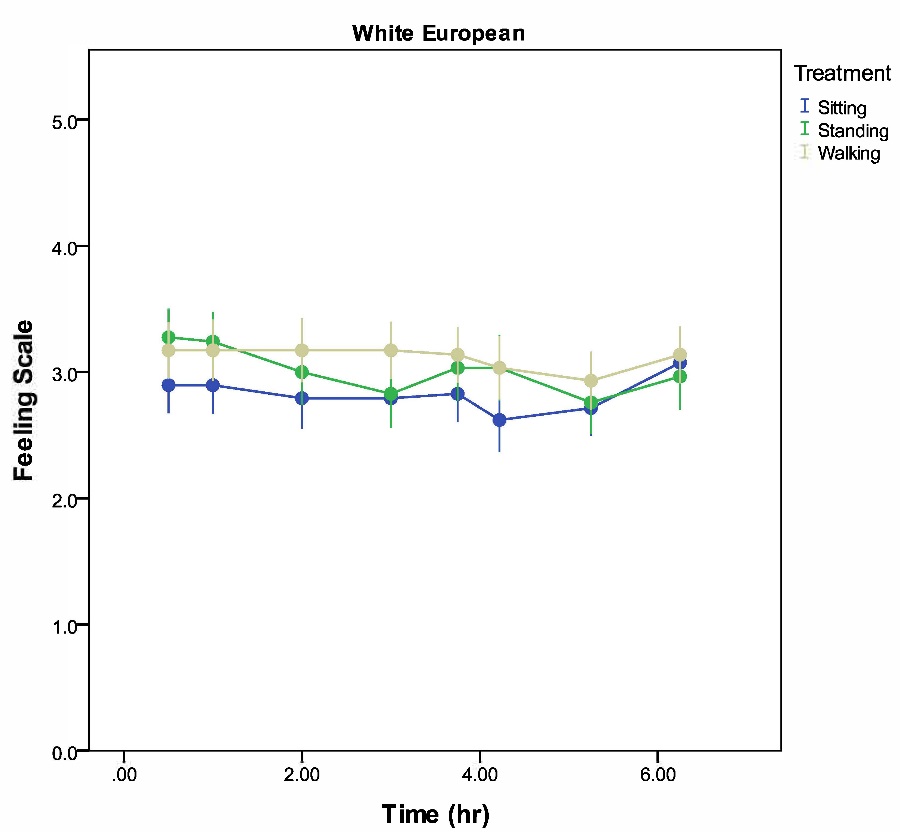

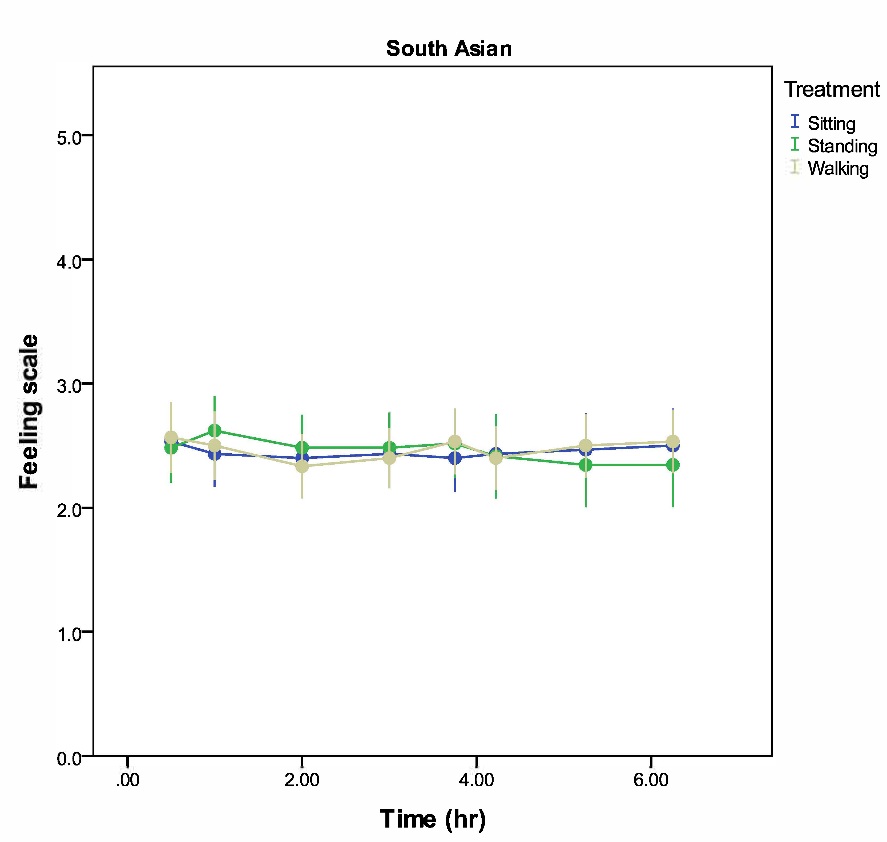


Error bars show the standard error within each condition at each time point
